# High-Accuracy Wave Direction Estimation Using Kalman Fusion of Interferometric Measurements and Energy Field Reconstruction

**DOI:** 10.3390/s26061852

**Published:** 2026-03-15

**Authors:** Caicheng Wang, Xue Li, Linshan Xue

**Affiliations:** 1 School of Microelectronics and Communication Engineering, Chongqing University, Chongqing 400044, China; wccwil_be@163.com; 2 The Center of Communication and Tracking Telemetry Command, Chongqing University, Chongqing 400044, China; 3 China Academy of Space Technology (CAST), Beijing 100081, China

**Keywords:** microwave wireless power transfer, beam pointing estimation, interferometric angle measurement, energy-field reconstruction

## Abstract

**Highlights:**

We propose a high-accuracy wave-direction estimation framework that fuses interferometric phase-based angle measurements with energy-field reconstruction. We develop a multi-rate Kalman fusion strategy to improve robustness and precision over single-source methods, validated under varying SNR and multi-target scenarios.

**What are the main findings?**

- A high-accuracy wave-direction estimation framework is developed by fusing interferometric phase-based angle measurements with energy-field reconstruction, exploiting their complementary strengths.
- A multi-rate Kalman fusion strategy effectively integrates high-rate interferometric observations and low-rate energy-based constraints, achieving improved accuracy and robustness under low SNR and multi-target conditions.

**What are the implications of the main findings?**

- The proposed fusion paradigm provides a practical and generalizable solution for reliable direction estimation in array-based remote sensing and electromagnetic sensing systems operating in noisy and complex environments.
- The proposed method can reduce sensitivity to measurement noise and scene complexity, supporting more stable pointing for applications such as microwave sensing links and space-based remote measurement scenarios.

**Abstract:**

Microwave wireless power transfer (MWPT) for space solar power stations (SSPS) requires high-precision beam pointing in order to maintain effective aperture coupling and transmission efficiency under platform motion and disturbances. This paper proposes a dual-link beam pointing estimation framework that integrates guidance-link interferometric angle-of-arrival (AoA) measurements with power-link energy-field reconstruction. The interferometric chain provides high-rate azimuth and elevation observations for dynamic tracking, while the energy-field reconstruction estimates the energy-centroid displacement from the received-aperture power distribution to correct steady-state pointing bias. A Kalman filter (KF) is developed to fuse the asynchronous multi-rate measurements, yielding continuous and robust pointing estimates for closed-loop beam control. Simulation results show that the proposed fusion method achieves azimuth and elevation RMSEs of $0.0069^{{}^{\circ}}$ and $0.006^{{}^{\circ}}$ with interferometric and energy-centroid error levels of approximately $0.05^{{}^{\circ}}$ and $0.02^{{}^{\circ}}$, respectively, significantly reducing high-frequency fluctuations. In addition, a sensitivity model is established to quantify the impact of angular errors on capture efficiency. The expected efficiency improves from approximately 0.988 and 0.998 for the individual methods to nearly unity for the fusion output. Quantitative accuracy thresholds corresponding to different efficiency requirements are further derived, providing practical guidelines for SSPS MWPT system design.

## 1. Introduction

Microwave wireless power transfer (MWPT) is widely regarded as a key technology enabling long-distance energy delivery in space solar power system (SSPS) architectures. In a typical SSPS configuration, solar energy collected in space is converted into microwave radiation and transmitted to the ground, where a rectifying antenna (rectenna) array converts the received beam into electrical power. Therefore, the feasibility and engineering viability of SSPS systems are jointly determined by transmission efficiency and beam-pointing capability [1,2].

In MWPT links, maximizing the received power requires the transmitted beam to be highly directive as well as accurately aligned with the receiving aperture. Consequently, beam control and pointing accuracy become key factors affecting system performance [3,4]. Phased-array beamforming and scanning technologies remain the primary approaches for achieving high directivity and flexible beam control in MWPT systems [5]. For long-range energy transmission, phased-array systems with wide-angle scanning capability can significantly improve system availability and adaptability to receiver position variations [6]. Experimental studies have also demonstrated the feasibility of high-precision beam control in phased-array MWPT systems [7,8].

However, beam alignment accuracy in SSPS systems is influenced not only by array control strategies but also by platform motion, attitude disturbances, and structural errors. Under non-ideal orbital configurations or scan angle variations, the received power may fluctuate with changing relative geometry, which requires beam-pointing measurements to exhibit strong dynamic adaptability [9]. Moreover, MWPT link efficiency is closely related to the coupling state of the energy beam within the receiving aperture, making accurate estimation of beam direction essential for maintaining stable energy transfer [10].

Existing studies on SSPS microwave transmission technologies have investigated array construction, beam control, and alignment mechanisms [11,12,13]. At the array implementation level, phased-array design and amplitude–phase control strategies have been widely explored to improve field distribution accuracy and pointing stability [14,15,16]. However, alignment methods based solely on angular measurements may not fully characterize the actual displacement of the energy beam on the receiving plane. Therefore, reconstructing the energy field distribution provides an additional measurement dimension for beam-pointing estimation [17,18].

To address this limitation, bidirectional beam-pointing measurement approaches combining interferometric angle measurements with energy-field reconstruction have been proposed for SSPS applications [19]. These methods estimate beam-pointing errors by jointly utilizing phase difference information and spatial power distribution on the receiving plane, enabling more accurate alignment correction. Recent experimental demonstrations have also verified the feasibility of high-precision estimation of the beam direction in practical MWPT systems [20,21,22].

Building upon these studies, this paper proposes a dual-link direction estimation framework for SSPS MWPT systems. The proposed method integrates interferometric angle-of-arrival (AoA) measurements with energy field distribution reconstruction to achieve robust and high-precision beam-pointing estimation. Specifically, interferometric processing provides angle measurements with a high update rate for dynamic tracking, while energy-centroid estimation derived from the received power distribution offers accurate steady-state correction of the pointing bias. These heterogeneous observations are fused using a Kalman filtering framework to support closed-loop beam control.

The main contributions of this paper are summarized as follows:

- A dual-link beam-direction estimation framework is proposed for SSPS MWPT systems, integrating time-modulated interferometric AoA measurements with energy field reconstruction to enable robust beam-pointing estimation under dynamic conditions.
- A hybrid measurement mechanism is developed in which interferometric observations with a high update rate support dynamic beam tracking, while energy-centroid estimation based on received-aperture power sampling provides accurate steady-state pointing correction.
- A Kalman filter-based fusion strategy is designed to integrate the two heterogeneous measurements and support closed-loop beam control, improving pointing accuracy and transmission efficiency in the presence of multiple error sources.

## 2. System Architecture and Methodology

### 2.1. System Model and Efficiency Analysis

In space-based MWPT, a phased array transmitter forms a directive energy beam to illuminate a receiving rectenna aperture. The end-to-end energy utilization is highly sensitive to beam pointing errors; angular misalignment with respect to the aperture normal or lateral displacement of the beam center distorts the power distribution on the receiving plane and causes edge-truncation loss, reducing the effectively received power. Hence, real-time and high-precision estimation of pointing deviations is essential for closed-loop beam alignment under platform motion and attitude disturbances.

**Figure 1:**
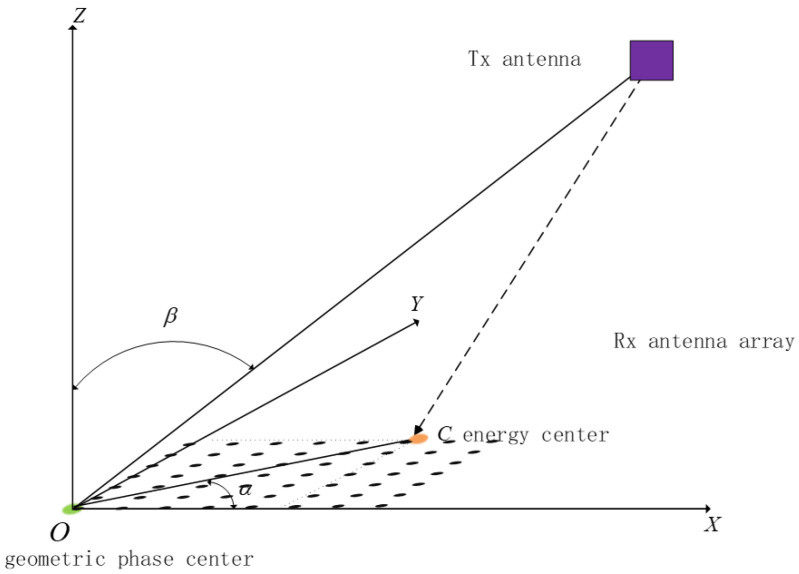
System geometry and angle definitions for space-based microwave wireless power transfer.

To unify the angle definitions used in subsequent interferometric estimation and energy-centroid inversion, a local coordinate system Σ: *O*–xyz is established on the receiving aperture (Figure 1). The origin *O* is located at the geometric center of the aperture, and the *z*-axis is aligned with the aperture normal. The incident direction is characterized by the azimuth angle α and elevation angle β with the unit direction vector:

(1) $$u\left(\alpha ,\beta \right)=\left[\begin{array}{c} \sin\beta \cos\alpha \\ \sin\beta \sin\alpha \\ \cos\beta \end{array}\right].$$

The system operates at carrier frequency $f_{c}$ with wavelength $\lambda =c/f_{c}$, and the transmitter–receiver separation is *D*. The transmission aperture applies amplitude tapering to suppress sidelobes; a truncated Gaussian model is adopted with

(2) $$A\left(r\right)=\exp\;\left(-a{\left(\frac{r}{R_{t}}\right)}^{2}\right),\;a=\ln\;\left(10^{T_{t}/20}\right),$$

where *r* is the radial distance from the aperture center, $R_{t}$ is the equivalent aperture radius, and $T_{t}$ is the edge-taper factor (dB).

Modeling the transmitting array as a coherent superposition of *N* elements with complex excitation $a_{i}e^{j\varphi_{i}}$, the received field at point r on the receiving plane is approximated as

(3) $$E\left(r\right)\approx \sum_{i=1}^{N}a_{i}\exp\;\left(j\left(kR_{i}\left(r\right)+\varphi_{i}\right)\right),$$

where k=2π/λ and $R_{i}\left(r\right)$ denotes the propagation distance. The power density is

(4) $$\Psi \left(r\right)=\frac{1}{\eta }{\left|E\left(r\right)\right|}^{2},$$

where η denotes the intrinsic impedance of free space, and the total received power over the receiving region $\Omega_{r}$ is

(5) $$P_{r}={\;\int \int \;}_{\Omega_{r}}\Psi \left(r\right)\;d\Omega .$$

When the beam is well-aligned and centered near *O*, Ψ(r) is approximately centro-symmetric and $P_{r}$ is maximized. Pointing errors or beam-center displacement shift the peak of the main lobe toward the aperture edge and increase the truncation loss, leading to a reduction in both $P_{r}$ and overall transfer efficiency.

### 2.2. Bidirectional Angle Estimation Framework

**Figure 2:**
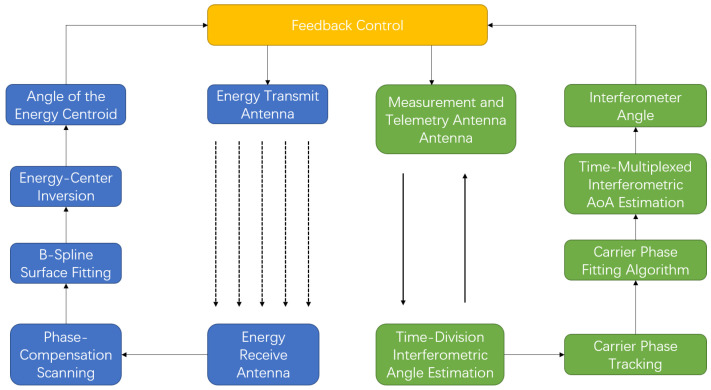
Overall processing flow of the proposed bidirectional angle estimation framework.

To simultaneously achieve rapid response and stable accuracy under highly dynamic conditions, this paper proposes a bidirectional angle estimation framework that integrates interferometric angle measurement with energy field-based angle inversion. By fusing measurements from the two complementary links through filtering-based data fusion, continuous angle estimates suitable for beam control are obtained. The overall processing flow of the proposed framework is illustrated in Figure 2.

By tracking the carrier phase of the guiding signal across multiple antennas, baseline phase differences can be formed to enable interferometric direction-finding. Let $b_{ij}$ denote the baseline vector of the *i*-th antenna pair and let u(α,β) denote the incident direction. The geometric phase difference is modeled as

(6) $$\Delta \phi_{ij}=\frac{2\pi }{\lambda }\;b_{ij}^{T}u+2\pi N_{ij}+\varepsilon_{ij},$$

where $N_{ij}$ is the integer ambiguity and $\varepsilon_{ij}$ accounts for phase noise and hardware-induced errors. A multi-baseline strategy is adopted in which short baselines provide ambiguity-free coarse estimates while long baselines yield higher angular resolution after ambiguity resolution. The detailed reconstruction and robust ambiguity resolution procedures are presented in subsequent sections.

In parallel, the actual beam offset on the receiving aperture is characterized by the measured power distribution. Discrete power samples $\left\{P_{m}\right\}$ are collected and used to reconstruct a continuous distribution $\hat{\Psi }\left(x,y\right)$ via interpolation or surface fitting, from which the energy centroid $\left(x_{c},y_{c}\right)$ is estimated. The centroid displacement relative to the aperture center *O* is

(7) $$d=\sqrt{x_{c}^{2}+y_{c}^{2}}.$$

Given the transmission distance *D*, the beam pointing deviation can be approximated under small-angle conditions as follows:

(8) $$\delta \approx \arctan\;\left(\frac{d}{D}\right)\approx \frac{d}{D}.$$

These two measurement modalities are complementary and operate at different time scales. Interferometric measurements provide high update rates but are sensitive to phase noise and ambiguity resolution, whereas energy-centroid measurements directly reflect beam offset but update more slowly. To exploit this complementarity, a unified state-space model and a filtering-based fusion framework are adopted to integrate the asynchronous measurements and generate robust angle estimates for beam control. The fused estimates are then fed back to drive transmitter beam pointing, enabling closed-loop alignment and improved power transfer efficiency [23].

## 3. Interferometric Angle Estimation Based on Time-Division Processing

### 3.1. Phase Difference-Based Interferometric Angle Estimation

Interferometric direction finding estimates the direction of arrival (DoA) from the spatial carrier phase difference measured by a baseline array. For a two-antenna baseline with spacing *d* under far-field conditions, the phase difference satisfies [24]:

(9) $$\Delta \varphi =\frac{2\pi d}{\lambda }\sin\theta$$

and the DoA can be obtained as follows:

(10) $$\theta =\arcsin\;\left(\frac{\lambda }{2\pi d}\Delta \varphi \right)$$

When the baseline is long (d>λ/2), the measured phase is wrapped into (−π,π], which introduces an integer-cycle ambiguity [25]. To achieve both ambiguity-free operation and high accuracy, a dual-baseline configuration is adopted.

**Figure 3:**
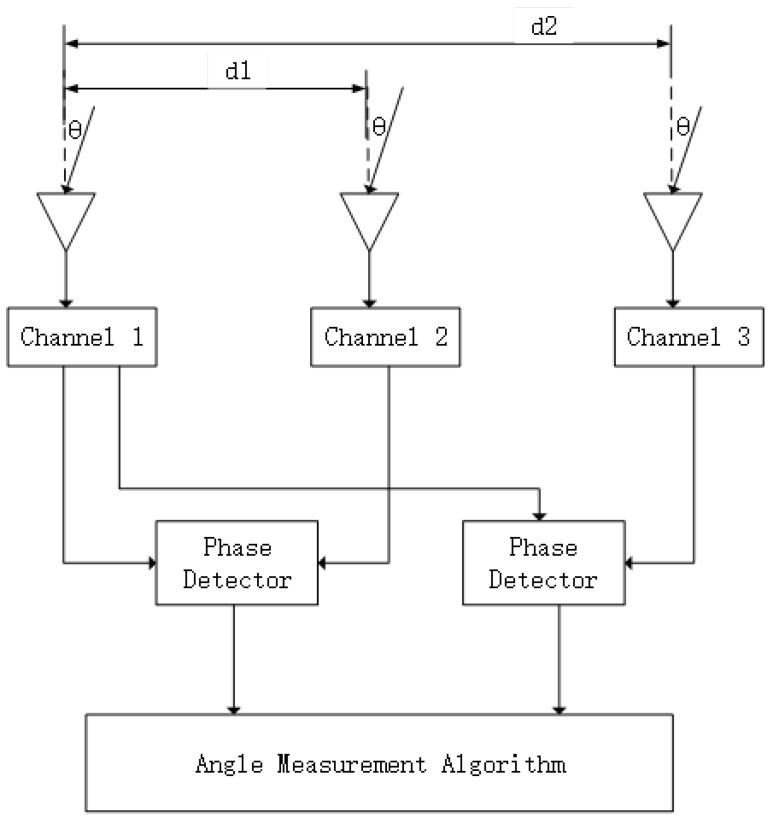
Dual-baseline interferometric angle estimation architecture.

As illustrated in Figure 3, a three-antenna interferometer forms a short baseline $d_{1}$ and a long baseline $d_{2}$. The phase differences from the two antenna pairs are extracted by phase detectors and jointly processed in the angle estimation module, where the short-baseline output provides a coarse ambiguity-free constraint and the long-baseline output is used for high-precision estimation after ambiguity resolution [26,27].

For the two baselines, the wrapped phase differences are $\Delta \varphi_{1},\Delta \varphi_{2}\in \left(-\pi ,\pi \right]$ and the corresponding true phases are modeled as

(11) $$\Delta \varphi_{1}+2\pi N_{1}=\frac{2\pi d_{1}}{\lambda }\sin\theta ,$$

(12) $$\Delta \varphi_{2}+2\pi N_{2}=\frac{2\pi d_{2}}{\lambda }\sin\theta ,$$

where $N_{1},N_{2}\in Z$ are the integer ambiguities [28]. A search-based ambiguity resolution is employed by traversing candidate $N_{1}$ within a feasible set and computing the implied real-valued ambiguity of the long baseline. The optimal ambiguity is selected using a nearest-integer consistency metric:

(13) $$J\left(N_{1}\right)=\left|{\hat{N}}_{2}\left(N_{1}\right)-\mathrm{round}\;\left({\hat{N}}_{2}\left(N_{1}\right)\right)\right|.$$

The integer ambiguity $N_{1}$ is searched within a feasible set determined by the expected phase difference range under the system geometry, and the optimal solution is selected by minimizing the consistency metric defined in (13). The ambiguity-free DoA is finally obtained by substituting $\left(N_{1}^{*},N_{2}^{*}\right)$ back into (12) and (13), using the long-baseline result for the final high-accuracy angle output.

### 3.2. Time-Modulated Interferometric Angle Estimation System Design

Conventional interferometric angle estimation is typically designed for continuous reception of a single target. When extended to multi-target service, simultaneous reception may cause signal superposition and phase-source confusion, which violates the assumption of phase consistency required for interferometric processing and degrades DoA estimation. To address this issue without introducing additional RF chains or complex multiplexing, a time-modulated reception scheme is adopted to separate targets in the time domain.

**Figure 4:**
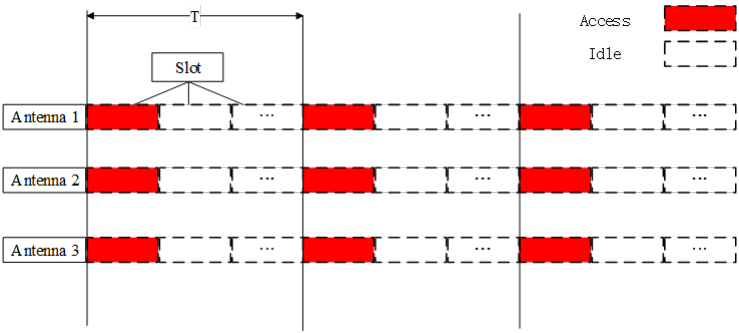
Time-modulated reception mechanism for multi-target separation in interferometric angle estimation.

As illustrated in Figure 4, the time axis is partitioned into periodic frames with duration *T*, and each frame is further divided into synchronized reception slots. In the slot assigned to a target, all antennas are activated simultaneously to acquire one snapshot of carrier-phase observations for that target; outside the slot, reception is disabled to suppress non-target signals and noise. By allocating non-overlapping slots to different targets, independent phase observation sequences can be formed for each target, enabling multi-target discrimination within a single interferometric receiver.

To facilitate subsequent carrier-phase reconstruction and interferometric processing, an equivalent signal model with an explicit gating formulation is established. Consider an *N*-antenna receiving array and *M* targets. Let $s_{m}\left(t\right)$ denote the complex baseband waveform of the *m*-th guiding signal (including spreading/data modulation), and let $A_{m}$, $f_{d,m}$, and $\phi_{i,m}$ denote the received amplitude, Doppler shift, and carrier phase at the *i*-th antenna, respectively. The received passband signal from the *m*-th target at the *i*-th antenna is

(14) $$r_{i,m}\left(t\right)=ℜ\;\left\{A_{m}\;s_{m}\;\left(t-\tau_{i,m}\right)e^{j\left(2\pi \left(f_{c}+f_{d,m}\right)t+\phi_{i,m}\right)}\right\}+n_{i}\left(t\right),$$

where $\tau_{i,m}$ is the equivalent propagation delay and $n_{i}\left(t\right)$ denotes noise and interference.

Define a unit rectangular window of width τ as

(15) $$w_{\tau }\left(t\right)=\left\{\begin{array}{cc} 1, & 0\leq t<\tau ,\\ 0, & \mathrm{otherwise}, \end{array}\right.$$

and define the periodic gating function for the *m*-th target with slot offset $t_{m}\in \left[0,T\right)$ as

(16) $$g_{m}\left(t\right)=\sum_{k=-\infty }^{+\infty }w_{\tau }\;\left(t-kT-t_{m}\right).$$

With non-overlapping slot scheduling, at most one gating function is active at any time instant. Therefore, the equivalent time-slotted received signal at the *i*-th antenna is

(17) $$x_{i}\left(t\right)=\sum_{m=1}^{M}g_{m}\left(t\right)\;r_{i,m}\left(t\right).$$

After downconversion and low-pass filtering, the complex baseband representation can be expressed as follows:

(18) $$y_{i}\left(t\right)=\sum_{m=1}^{M}g_{m}\left(t\right)\;A_{m}\;s_{m}\;\left(t-\tau_{i,m}\right)e^{j\left(2\pi f_{d,m}t+\phi_{i,m}\right)}+w_{i}\left(t\right)$$

where $w_{i}\left(t\right)$ is the complex baseband noise term. Since the slots assigned to different targets do not overlap, the baseband signal contains at most one target at any time, avoiding superposition and preserving within-slot phase consistency for interferometric measurements.

Within each active slot, standard correlation-based acquisition/tracking is performed to obtain carrier-phase observations for the designated target. Following established robust carrier-phase tracking designs for multi-peak correlation signals (e.g., BOC-type waveforms) [29], the receiver output is abstracted as carrier-phase measurements with bounded noise. Consequently, for each target, the carrier-phase observations form a periodically sampled discrete-time sequence. During idle intervals, reception is disabled and the carrier numerically controlled oscillator (NCO) may free-run for prediction. Based on these discrete observations, the subsequent section reconstructs continuous-time carrier-phase trajectories (via fitting or state-space modeling), enabling time-aligned baseline phase differences and robust ambiguity-resolved interferometric angle estimation.

### 3.3. Carrier Phase Fitting Algorithm

Under the time-slotted reception mechanism, the acquisition and tracking loops output a carrier-phase observation only once within each slot of a given period, while no valid correlation accumulation is available during idle intervals. Therefore, as described in Section 3.2, the carrier-phase observations are periodically discrete in time and can be expressed as

(19) $${\hat{\phi }}_{i}\left[k\right]={\hat{\phi }}_{i}\left(kT\right),$$

where *k* denotes the period index, *T* is the time-slotted reception period, and ${\hat{\phi }}_{i}\left[k\right]$ represents the carrier-phase observation obtained from the carrier tracking channel during the *k*-th period. Since interferometric angle estimation requires constructing continuous-time baseline phase differences and further performing ambiguity resolution and filtering, it is necessary to expand the discrete carrier-phase observations into a continuous phase trajectory. This ensures temporal alignment of phase differences and provides phase predictions during idle intervals. To this end, a carrier-phase fitting algorithm based on sliding-window polynomial modeling is proposed.

The carrier-phase output of the tracking loop is typically wrapped modulo 2π, confined to the interval [−π,π) or [0,2π). Prior to fitting, phase unwrapping must be applied to the discrete phase sequence in order to obtain a continuous phase series:

(20) $$\phi_{i}\left[k\right]=\mathrm{unwrap}\;\left({\hat{\phi }}_{i}\left[k\right]\right).$$

Phase unwrapping can be implemented using phase-difference detection. Let the phase increment between consecutive periods be defined as follows:

(21) $$\Delta {\hat{\phi }}_{i}\left[k\right]={\hat{\phi }}_{i}\left[k\right]-{\hat{\phi }}_{i}\left[k-1\right].$$

If $|\Delta {\hat{\phi }}_{i}\left[k\right]|>\pi$, then an integer multiple of 2π is added or subtracted to enforce phase continuity, yielding the unwrapped phase $\phi_{i}\left[k\right]$.

In space-based wireless power transfer scenarios involving highly dynamic relative motion between satellites and ground platforms, the temporal variation of carrier phase is primarily governed by Doppler shifts and their rates of change. Over a relatively short time window, the phase evolution can be approximated well by a low-order polynomial. Accordingly, this work adopts a sliding-window polynomial fitting approach in order to locally approximate the continuous carrier-phase trajectory. Taking the *k*-th period as the current reference, the phase within a window of length *P* can be modeled as

(22) $$\phi_{i}\left(t\right)\approx a_{i,0}+a_{i,1}\left(t-t_{k}\right)+a_{i,2}{\left(t-t_{k}\right)}^{2}+\cdots +a_{i,P}{\left(t-t_{k}\right)}^{P},$$

where $t_{k}=kT$, $a_{i,p}$ are the polynomial coefficients and *P* denotes the polynomial order. To balance dynamic adaptability and numerical stability, second- or third-order polynomials are recommended. A second-order polynomial is suitable for windows with approximately constant acceleration, while a third-order polynomial can further accommodate variations in acceleration.

Within the sliding window, discrete phase samples are taken at times

(23) $$t_{k-m}=\left(k-m\right)T,\;m=0,1,\ldots ,K-1,$$

corresponding to the unwrapped phase observations $\phi_{i}\left[k-m\right]$. The associated design matrix is constructed as follows:

(24) $$X=\left[\begin{array}{ccccc} 1 & \Delta t_{0} & \Delta t_{0}^{2} & \cdots & \Delta t_{0}^{P}\\ 1 & \Delta t_{1} & \Delta t_{1}^{2} & \cdots & \Delta t_{1}^{P}\\ \vdots & \vdots & \vdots & \ddots & \vdots \\ 1 & \Delta t_{K-1} & \Delta t_{K-1}^{2} & \cdots & \Delta t_{K-1}^{P} \end{array}\right]$$

where $\Delta t_{m}=t_{k-m}-t_{k}=-mT$. The corresponding observation vector is then formed as

(25) $$y_{i}=\left[\begin{array}{c} \phi_{i}\left[k\right]\\ \phi_{i}\left[k-1\right]\\ \vdots \\ \phi_{i}\left[k-K+1\right] \end{array}\right].$$

The polynomial coefficients can then be estimated using the weighted least-squares method as

(26) $${\hat{a}}_{i}={\left(X^{T}WX\right)}^{-1}X^{T}Wy_{i},$$

where W denotes the weighting matrix. If the variances of the carrier-phase observations are approximately identical, W can be set to the identity matrix I. When the carrier-phase noise levels differ significantly among slots from different periods, the weights can be chosen inversely proportional to the corresponding phase-noise variances, thereby improving the robustness of the fitting process under weak-signal conditions.

After the coefficient vector ${\hat{a}}_{i}$ is obtained, the carrier phase can be reconstructed at any continuous time instant *t* as

(27) $${\tilde{\phi }}_{i}\left(t\right)=\sum_{p=0}^{P}{\hat{a}}_{i,p}{\left(t-t_{k}\right)}^{p}.$$

When *t* falls within the idle interval of the current period, ${\tilde{\phi }}_{i}\left(t\right)$ represents the predicted carrier phase for the unobserved time segment, thereby filling the phase gaps introduced by time-slotted reception. To ensure consistency with subsequent interferometric phase-difference construction, a modulo-2π normalized phase output can be computed as

(28) $${\tilde{\phi }}_{i}^{\mathrm{mod}}\left(t\right)=\mathrm{wrap}\;\left({\tilde{\phi }}_{i}\left(t\right)\right).$$

Furthermore, to guarantee phase alignment across different channels, the same reference period index and same prediction time instant *t* are adopted for all channels during phase reconstruction. In this work, carrier-phase fitting and reconstruction are performed independently for each channel, followed by baseline phase-difference construction at a unified time instant. This step is critical to the overall method, as it ensures that baseline phase differences are constructed using phase estimates corresponding to the same physical time even under time-slotted reception. Consequently, reliable interferometric phase differences can be formed and used for subsequent ambiguity resolution and angle estimation.

To mitigate the impact of fitting instability on phase-difference accuracy, practical implementations may adopt the same polynomial order *P* and window length across all channels. In addition, when the number of valid observations is insufficient or the proportion of abnormal measurements becomes excessive, adaptive strategies such as weight reduction or window shortening can be triggered to maintain continuity and reliability of the reconstructed phase sequences.

## 4. Multi-Source Fusion High-Precision Angle Estimation Based on Kalman Filtering

### 4.1. Overall Framework of the Fusion Angle Estimation Algorithm

As described in Section 3.2, this work establishes the acquisition and tracking chains for BOC signals and constructs an equivalent signal model under time-slotted reception. To address the discontinuity of carrier-phase observations introduced by time-slotted reception, a sliding-window polynomial fitting algorithm is employed to reconstruct continuous carrier-phase trajectories for each channel. Baseline phase-difference sequences are then constructed at unified time instants. Based on the reconstructed continuous baseline phase differences, short-baseline ambiguity-free interferometric angle estimation and long-baseline high-precision angle estimation with ambiguity resolution are jointly applied to obtain the interferometric angle outputs, which can be expressed as a sequence of azimuth and elevation measurements:

(29) $$z_{k}^{\left(I\right)}=\left[\begin{array}{c} \alpha_{k}^{\left(I\right)}\\ \beta_{k}^{\left(I\right)} \end{array}\right]$$

where *k* denotes the filter update index.

On the other hand, the measurement based on the energy centroid derives pointing angle information from the power distribution over the receiving aperture or from fitted energy-beam surfaces. This measurement can also be represented as a set of azimuth and elevation observations:

(30) $$z_{k}^{\left(E\right)}=\left[\begin{array}{c} \alpha_{k}^{\left(E\right)}\\ \beta_{k}^{\left(E\right)} \end{array}\right].$$

Compared with interferometric measurements, observations based on the energy centroid typically have a lower update rate but exhibit smaller long-term drift. As such, they can be used to provide low-frequency correction and steady-state constraints for interferometric angle estimates. Although the energy-centroid measurement is obtained after phase compensation based on the interferometric estimate, the dominant error sources of the two measurements are fundamentally different. Interferometric observations are mainly affected by carrier-phase noise and ambiguity resolution errors, whereas the energy-centroid measurement is determined by reconstructing the spatial power distribution over the receiving aperture. Therefore, the correlation between the two measurement noises is considered negligible in practice, and the covariance matrices $R_{I}$ and $R_{E}$ are modeled as independent in the Kalman filtering framework.

Accordingly, this work treats interferometric angle measurements as high-rate observations and measurements based on the energy centroid as low-rate but high-accuracy correction observations. A unified state-space model is formulated, with a Kalman filter–based multi-source fusion strategy adopted to combine the two measurement types. Under highly dynamic conditions, this approach enables smooth, continuous, and high-precision angle estimation. The fused angle estimates can be directly used for beam steering and closed-loop pointing control, and also provide reliable constraints for subsequent ambiguity resolution and carrier-phase continuity validation.

### 4.2. Energy Centroid-Based Measurement

#### 4.2.1. Power Sensing Array and Coordinate System Definition

A power sensing array is deployed on the receiving aperture Π, the normal direction of which is denoted by n. A two-dimensional coordinate system $O_{xy}$ is established on the aperture plane, where *O* denotes the geometric center of the aperture; the *x*- and *y*-axes lie in the plane and are mutually orthogonal. The array consists of $N_{p}$ power-sensing elements. The position of the *q*-th sensing element in the aperture coordinate system is given by

(31) $$r_{q}=\left[\begin{array}{c} x_{q}\\ y_{q} \end{array}\right],$$

where $q=1,2,\ldots ,N_{p}$.

For a given measurement epoch, the power sensing array provides a set of discrete power samples:

(32) $$S=\{\left(r_{q},P_{q}\right)|q=1,2,\ldots ,N_{p}\}$$

where $P_{q}$ denotes the measured power at the *q*-th sensing element.

Since the outputs of the power sensors are affected by measurement noise, quantization errors, and array non-idealities, the power measurement can be modeled as

(33) $$P_{q}=\bar{P}\left(x_{q},y_{q}\right)+n_{p,q},$$

where $\bar{P}\left(x,y\right)$ denotes the continuous power distribution over the aperture plane and $n_{p,q}$ represents the power measurement error.

The centroid of the energy beam in the aperture coordinate system is defined as the projection offset vector:

(34) $$c=\left[\begin{array}{c} x_{c}\\ y_{c} \end{array}\right].$$

When the beam center does not coincide with the aperture center, i.e., when c≠0, the objective of the energy centroid-based measurement is to estimate c and then further convert it into the corresponding azimuth and elevation observations.

#### 4.2.2. Power Distribution Interpolation and Fitting

Due to residual beam pointing errors, the energy beam projected onto the receiving aperture generally exhibits offset and non-uniform characteristics. If the energy centroid is directly estimated from discrete power samples, the result is susceptible to systematic biases induced by oblique incidence and pointing errors, thereby degrading measurement accuracy. To address this issue, in this work we adopt a phase-compensated scanning strategy combined with two-dimensional surface interpolation and fitting. By compensating for geometric phase errors, the power distribution is reshaped toward an ideal symmetric form, after which a continuous power surface is reconstructed from discrete samples to provide a high-resolution input for subsequent energy centroid estimation.

Under far-field conditions, the incident wavefront can be approximated as planar. Let the aperture coordinate system be defined as in Section 4.2.1. The position of the *i*-th array element in the aperture plane is denoted by $\left(x_{i},y_{i}\right)$, and the incident direction is described by the azimuth angle α and elevation angle β. The geometric propagation delay of the *i*-th element relative to the reference element can be expressed as

(35) $$\tau_{i}\left(\alpha ,\beta \right)=\frac{1}{c}\left(x_{i}\sin\beta \cos\alpha +y_{i}\sin\beta \sin\alpha \right),$$

where *c* denotes the speed of light. The corresponding geometric phase term is given by

(36) $$\phi_{i}\left(\alpha ,\beta \right)=\frac{2\pi }{\lambda }c\;\tau_{i}\left(\alpha ,\beta \right)=\frac{2\pi }{\lambda }\left(x_{i}\sin\beta \cos\alpha +y_{i}\sin\beta \sin\alpha \right),$$

where λ is the carrier wavelength. For clarity, the phase difference can also be expressed directly as a function of the spatial coordinates of the receiving point. This representation avoids possible ambiguity in the intermediate time-delay formulation and ensures dimensional consistency.

For a candidate incident direction (α,β), the complex exponential of the geometric phase is used as the phase-compensation weight:

(37) $$w_{i}\left(\alpha ,\beta \right)=e^{-j\phi_{i}\left(\alpha ,\beta \right)}.$$

By coherently combining the array signals with these compensation weights, the received power P(α,β) can be computed. When the candidate direction approaches the true incident direction, the geometric phase errors are effectively corrected, the combined output power is maximized, and the resulting power distribution becomes closer to the ideal symmetric shape, thereby achieving pointing-direction calibration.

To reduce computational complexity, prior angle estimates provided by interferometric measurements are used to constrain the scanning region to a local neighborhood. Specifically, the candidate set is defined as $\alpha \in [\alpha_{0}-\Delta \alpha ,\alpha_{0}+\Delta \alpha ]$ and $\beta \in [\beta_{0}-\Delta \beta ,\beta_{0}+\Delta \beta ]$, with angular step sizes $\delta_{\alpha }$ and $\delta_{\beta }$, respectively. The optimal scanning direction is obtained by

(38) $$\left(\hat{\alpha },\hat{\beta }\right)=\arg\max_{\alpha ,\beta }P\left(\alpha ,\beta \right).$$

After obtaining $\left(\hat{\alpha },\hat{\beta }\right)$, phase compensation is applied to the array signals using this direction, and the power samples from the sensing array are read out to form the input for subsequent surface interpolation and fitting.

Assume that the power sensing array provides $N_{p}$ discrete power samples on the aperture plane, denoted by ${\left\{\left(x_{n},y_{n}\right),p_{n}\right\}}_{n=1}^{N_{p}}$. Considering the effects of measurement noise, calibration errors, and environmental disturbances, direct grid-based interpolation may lead to local noise amplification and spurious oscillations. Therefore, a smoothing-oriented processing strategy is adopted in this work.

First, the power samples are normalized as follows:

(39) $${\tilde{p}}_{n}=\frac{p_{n}}{\max_{k}\left(p_{k}\right)}.$$

Next, the physical aperture coordinates are linearly mapped onto a normalized parameter domain (u,v)∈[0,1]×[0,1] as

(40) $$u=\frac{x-x_{\min}}{x_{\max}-x_{\min}},\;v=\frac{y-y_{\min}}{y_{\max}-y_{\min}}.$$

This mapping yields the parametric coordinates $\left(u_{n},v_{n}\right)$ corresponding to each power sample.

A two-dimensional B-spline surface is employed to model the continuous power density distribution [30]:

(41) $$S\left(u,v\right)=\sum_{i=0}^{I}\sum_{j=0}^{J}c_{ij}B_{i}^{\left(p\right)}\left(u\right)B_{j}^{\left(q\right)}\left(v\right)$$

where $c_{ij}$ are the control coefficients to be estimated and $B_{i}^{\left(p\right)}\left(\cdot \right)$ and $B_{j}^{\left(q\right)}\left(\cdot \right)$ denote the B-spline basis functions in the *u*- and *v*-directions, respectively. The spline orders *p* and *q* are typically chosen as cubic to balance smoothness and fitting capability. The B-spline basis functions are defined using the Cox–de Boor recursion formula. Owing to their local support property, B-splines can effectively suppress global oscillations induced by noise and improve fitting stability.

By stacking the normalized power samples into a vector form, the surface fitting problem can be written in a matrix form as

(42) $$\tilde{p}=Ac+\varepsilon ,$$

where $\tilde{p}={\left[{\tilde{p}}_{1},\ldots ,{\tilde{p}}_{N_{p}}\right]}^{T}$, c is the vector of control coefficients arranged in lexicographic order, A is the basis matrix constructed from the B-spline basis functions evaluated at $\left(u_{n},v_{n}\right)$, and ε represents the fitting residual.

The control coefficient vector is estimated by the least-squares criterion as

(43) $$\hat{c}=\arg\min_{c}{∥Ac-\tilde{p}∥}_{2}^{2}.$$

To further suppress local noise effects and enhance surface smoothness, a second-order difference penalty term can be introduced, leading to a regularized least-squares formulation [23,31,32]:

(44) $$\hat{c}=\arg\min_{c}\left({∥Ac-\tilde{p}∥}_{2}^{2}+\lambda {∥Dc∥}_{2}^{2}\right)$$

where D denotes the discrete difference operator matrix and λ is the smoothing weight that controls the tradeoff between fitting fidelity and smoothness.

After obtaining $\hat{c}$, the continuous power surface $\tilde{S}\left(u,v\right)$ can be reconstructed and inverse coordinate mapping can be applied to recover the continuous power density distribution $\tilde{P}\left(x,y\right)$ on the aperture plane. This reconstructed surface serves as a high-resolution input for subsequent energy centroid detection and angle estimation.

#### 4.2.3. Energy Centroid Position Estimation and Angle Conversion

Let the geometric center of the receiving aperture be denoted by *O*. A local two-dimensional Cartesian coordinate system Oxy is established on the aperture plane, with *O* coinciding with the aperture center. The energy centroid is denoted by $P_{c}$, for which tne coordinates in the aperture plane are $\left(x_{c},y_{c}\right)$. The lateral offset distance of the energy centroid relative to the aperture center is denoted by *d*, which has been defined in Equation (7).

Let the phase center of the transmitting aperture be denoted by $P_{0}$ and let the distance between the transmitting and receiving phase centers be *D*. Under far-field and small-angle assumptions, the vertical height of the energy beam axis relative to the aperture normal can be approximated by the geometric relationship d≪D. According to the geometric mapping relationship, the elevation angle corresponding to the energy centroid offset can be expressed as

(45) $$\beta^{\left(E\right)}=\arctan\;\left(\frac{d}{D}\right).$$

This elevation angle reflects the inclination of the energy beam axis relative to the aperture normal, and corresponds to the elevation angle definition adopted in this work. The azimuth angle is determined by the in-plane direction of the energy centroid offset. Consistent with the definition of the azimuth angle α, the azimuth angle estimate based on the energy centroid is given by

(46) $$\alpha^{\left(E\right)}=\arctan2\left(y_{c},x_{c}\right),$$

where the arctan2(·) function is used to ensure correct quadrant determination and continuous angle definition over the full angular domain.

Finally, the energy centroid-based measurement vector is constructed as follows:

(47) $$z_{k}^{\left(E\right)}=\left[\begin{array}{c} \alpha_{k}^{\left(E\right)}\\ \beta_{k}^{\left(E\right)} \end{array}\right].$$

which serves as a low-frequency and high-accuracy observation input for the subsequent Kalman filter-based fusion framework.

### 4.3. Kalman Filter-Based Fusion Model Construction

The previous sections have established two types of measurements that can be used for angle estimation fusion. The interferometric angle estimation chain outputs angular measurements $z_{k}^{\left(I\right)}$ at each update epoch, which are obtained from carrier-phase fitting under time-slotted reception, baseline phase-difference construction, and ambiguity-resolved interferometric angle estimation. The energy centroid-based measurement chain provides angular measurements $z_{k}^{\left(E\right)}$, which are derived from power distribution fitting and energy centroid position estimation. The two measurement chains are complementary in terms of update rate and long-term stability. Accordingly, this work adopts a Kalman filter-based dynamic model to achieve multi-source fusion, enabling continuous, smooth, and high-precision angle estimation under highly dynamic conditions.

Considering that the angular variation can be approximated by a constant-velocity model over short time intervals, the state vector is defined as follows:

(48) $$x_{k}=\left[\begin{array}{c} \alpha_{k}\\ \beta_{k}\\ {\dot{\alpha }}_{k}\\ {\dot{\beta }}_{k} \end{array}\right]$$

where $\alpha_{k}$ and $\beta_{k}$ denote the true azimuth and elevation angles, respectively, and ${\dot{\alpha }}_{k}$ and ${\dot{\beta }}_{k}$ represent the corresponding angular velocity states.

Let the filter update interval be denoted by *T*. The state transition equation is then given by

(49) $$x_{k}=Fx_{k-1}+w_{k-1},$$

where the state transition matrix F is defined as

(50) $$F=\left[\begin{array}{cccc} 1 & 0 & T & 0\\ 0 & 1 & 0 & T\\ 0 & 0 & 1 & 0\\ 0 & 0 & 0 & 1 \end{array}\right].$$

The process noise term $w_{k-1}$ accounts for unmodeled angular acceleration and external disturbances, and is assumed to be zero-mean Gaussian with covariance matrix Q. For engineering convenience, the process noise covariance can be constructed using angular acceleration noise intensities $q_{\alpha }$ and $q_{\beta }$, with the covariance matrices for the azimuth and elevation dimensions given by

(51) $$Q_{\alpha }=q_{\alpha }\left[\begin{array}{cc} T^{3}/3 & T^{2}/2\\ T^{2}/2 & T \end{array}\right],\;Q_{\beta }=q_{\beta }\left[\begin{array}{cc} T^{3}/3 & T^{2}/2\\ T^{2}/2 & T \end{array}\right].$$

These are assembled into a block-diagonal process noise covariance matrix as

(52) $$Q=\left[\begin{array}{cc} Q_{\alpha } & 0\\ 0 & Q_{\beta } \end{array}\right].$$

The interferometric angle measurement is defined as

(53) $$z_{k}^{\left(I\right)}=\left[\begin{array}{c} \alpha_{k}^{\left(I\right)}\\ \beta_{k}^{\left(I\right)} \end{array}\right]$$

and the energy centroid-based measurement is defined as

(54) $$z_{k}^{\left(E\right)}=\left[\begin{array}{c} \alpha_{k}^{\left(E\right)}\\ \beta_{k}^{\left(E\right)} \end{array}\right].$$

Both types of measurements provide direct observations of the angular states. Therefore, a unified measurement model with the same observation matrix can be employed:

(55) $$z_{k}=Hx_{k}+v_{k}\;H=\left[\begin{array}{cccc} 1 & 0 & 0 & 0\\ 0 & 1 & 0 & 0 \end{array}\right]$$

where $v_{k}$ denotes the measurement noise.

The interferometric measurement noise $v_{k}^{\left(I\right)}$ is assumed to follow a zero-mean Gaussian distribution with covariance matrix $R_{I}$, while the energy centroid-based measurement noise $v_{k}^{\left(E\right)}$ is also modeled as zero-mean Gaussian with covariance matrix $R_{E}$. In general, $R_{E}\leq R_{I}$, reflecting the higher steady-state accuracy of the energy centroid-based measurement.

The time update (prediction) equations of the Kalman filter are given by

(56) $${\hat{x}}_{k}^{-}=F{\hat{x}}_{k-1},$$

(57) $$P_{k}^{-}=FP_{k-1}F^{T}+Q,$$

where ${\hat{x}}_{k}^{-}$ and $P_{k}^{-}$ denote the predicted state estimate and predicted error covariance, respectively.

For measurement updates, the standard Kalman filter equations are applied. For any available measurement $z_{k}$, the corresponding noise covariance matrix R is selected according to the measurement type, and the Kalman gain is computed as

(58) $$K_{k}=P_{k}^{-}H^{T}{\left(HP_{k}^{-}H^{T}+R\right)}^{-1}.$$

The state estimate and covariance matrix are then updated as

(59) $${\hat{x}}_{k}={\hat{x}}_{k}^{-}+K_{k}\left(z_{k}-H{\hat{x}}_{k}^{-}\right),$$

(60) $$P_{k}=\left(I-K_{k}H\right)P_{k}^{-}.$$

Considering that energy centroid-based measurements are updated at a lower rate, a multi-rate update strategy is adopted in practical implementation. Specifically, at each update epoch, interferometric measurements are used to perform state updates, while energy centroid-based measurements are incorporated one time per multiple epochs to provide low-frequency and high-accuracy correction. This strategy effectively fuses multi-source observations without modifying the filter structure. When both measurements are available at the same update instant, sequential measurement updates are performed, with the interferometric observation processed first followed by the energy-centroid measurement.

The filter is initialized at the first update epoch, with the initial angle state $\left(\alpha_{0},\beta_{0}\right)$ obtained from either interferometric measurements or energy centroid-based measurements. The initial covariance $P_{0}$ is set as a diagonal matrix according to the initial uncertainty of the angular estimates. The measurement noise covariance $R_{I}$ can be estimated from the residual statistics of the interferometric angle estimation chain or from the residual errors obtained during ambiguity resolution using short- and long-baseline measurements. The measurement noise covariance $R_{E}$ can be determined from repeated energy-centroid measurements under static or quasi-static conditions. The process noise parameters $q_{\alpha }$ and $q_{\beta }$ are used to characterize the intensity of angular acceleration disturbances, and can be tuned based on orbital relative motion models or empirical data.

The final output of the fusion filter is given by

(61) $${\hat{x}}_{k}=\left[\begin{array}{c} {\hat{\alpha }}_{k}\\ {\hat{\beta }}_{k}\\ {\dot{\hat{\alpha }}}_{k}\\ {\dot{\hat{\beta }}}_{k} \end{array}\right],$$

where ${\hat{\alpha }}_{k}$ and ${\hat{\beta }}_{k}$ are the fused high-precision estimates of the azimuth and elevation angles, respectively, and ${\dot{\hat{\alpha }}}_{k}$ and ${\dot{\hat{\beta }}}_{k}$ are the corresponding angular velocity estimates. In the absence of measurements and phase-consistency validation, the angular velocity states can be utilized for subsequent short-term angle prediction in beam-pointing control. As a result the proposed fusion framework enhances system robustness under weak-signal conditions or degraded measurement scenarios.

## 5. Simulation

### 5.1. Simulation Parameter Design

The simulation settings are summarized in Table 1. Unless otherwise specified, the same array configuration, baseline setup, and algorithm parameters are used across all experiments to ensure fair comparison. Two representative relative-motion profiles are considered, namely, constant acceleration and sinusoidal acceleration, leading to time-varying Doppler typical of SSPS beam-pointing scenarios. For statistical performance evaluation, Monte Carlo simulations with independent noise realizations are conducted (100 runs when RMSE curves are reported).

To improve the reproducibility of the numerical experiments, the main modeling parameters used in the Kalman fusion simulations are further summarized in Table 2, including the target number, noise assumptions, update rates, and the covariance settings adopted in the state-space model.

Unless otherwise specified, the interferometric subsystem adopts a three-antenna dual-baseline configuration, and the fusion simulations are performed under a single-target condition.

### 5.2. Validation of Guidance-Link Phase Observations and Reconstruction

**Figure 5:**
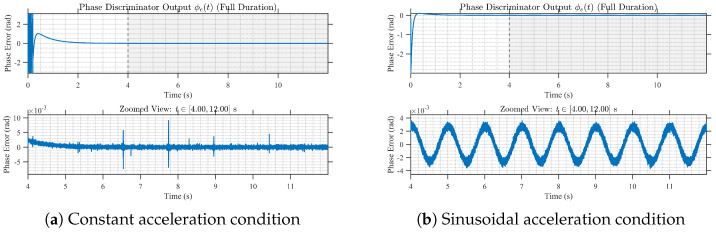
Time series comparison of carrier phase tracking under different dynamic conditions.

**Figure 6:**
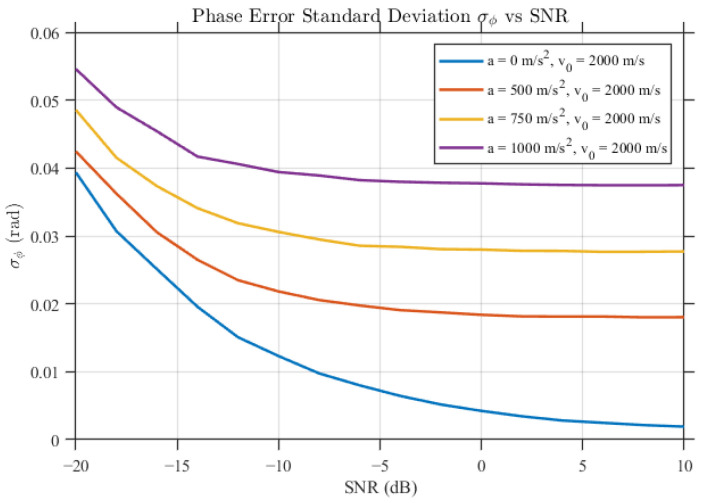
Variation of carrier phase error, showing standard deviation $\sigma_{\phi }$ vs. SNR.

Figure 5 illustrates representative carrier-phase tracking results under the two motion profiles. The tracking loop converges rapidly and remains stable during the observation interval, with steady-state residual phase errors staying at the milliradian level and no sustained divergence or loss of lock observed. To quantify the influence of SNR and dynamic stress, Figure 6 reports the standard deviation of carrier-phase errors, denoted $\sigma_{\phi }$, versus SNR. In low-to-moderate dynamic conditions, $\sigma_{\phi }$ decreases noticeably as SNR increases; under strong dynamics, the achievable phase accuracy becomes primarily limited by dynamic stress rather than SNR. Under time-slotted reception, the carrier-phase outputs are periodically sampled, and consequently require reconstruction prior to interferometric processing.

**Figure 7:**
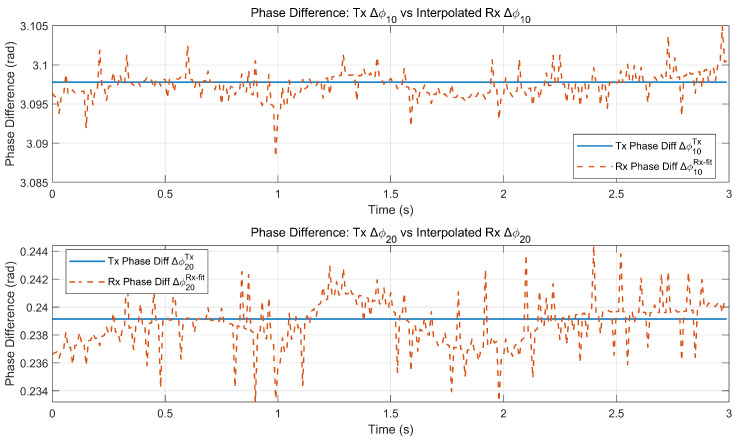
Comparison of reconstructed receiving-end and transmitting-end baseline phase differences after interpolation and fitting.

**Figure 8:**
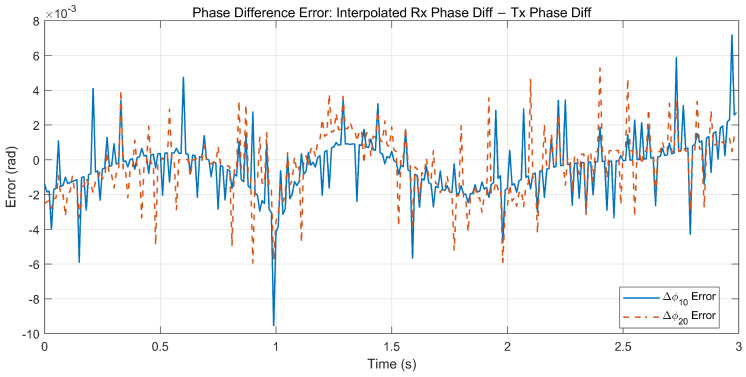
Error curves of reconstructed baseline phase differences.

Figure 7 and Figure 8 validate the proposed interpolation-based reconstruction: the reconstructed baseline phase differences closely follow the reference trends, and the reconstruction errors remain zero-mean with small fluctuations and no cycle slip-like jumps. These results confirm that the phase reconstruction step provides time-aligned and continuous-baseline phase difference inputs for the subsequent ambiguity-resolved interferometric angle estimation.

### 5.3. Performance Analysis of Interferometric Angle Estimation

**Figure 9:**
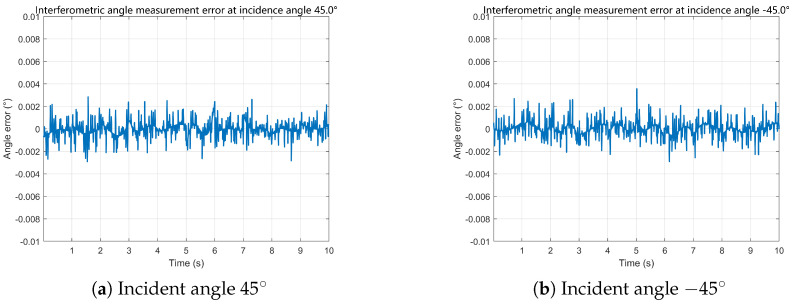
Interferometric angle measurement errors under constant acceleration conditions.

**Figure 10:**
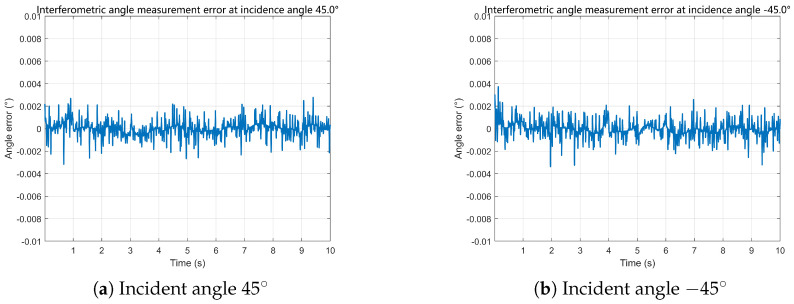
Interferometric angle measurement errors under sinusoidal acceleration conditions.

Based on the reconstructed baseline phase differences, the proposed time-modulated single-channel multi-baseline interferometric estimator is evaluated under different motion profiles. The representative time-domain angle error sequences shown in Figure 9 and Figure 10 fluctuate around zero without observable bias accumulation or long-term drift, indicating stable tracking performance under time-slotted sampling.

**Figure 11:**
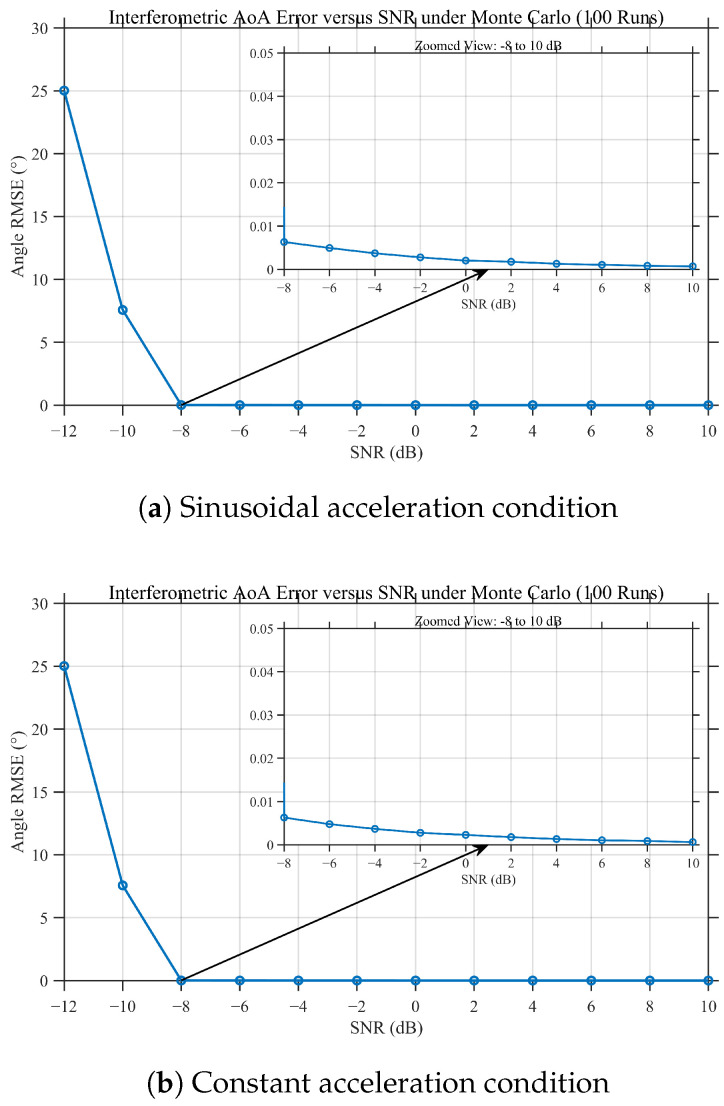
Monte Carlo simulation results of interferometric angle estimation RMSE vs. SNR under different dynamic conditions (100 runs).

Figure 11 presents the Monte Carlo RMSE results as a function of SNR obtained after 100 independent trials. A clear operating threshold is observed near SNR −8 dB. Below this threshold, ambiguity-related failures occur more frequently and the RMSE increases sharply; above the threshold, the estimator operates in a stable regime and achieves an RMSE on the order of $10^{-2}$, with gradual improvement as SNR increases.

**Figure 12:**
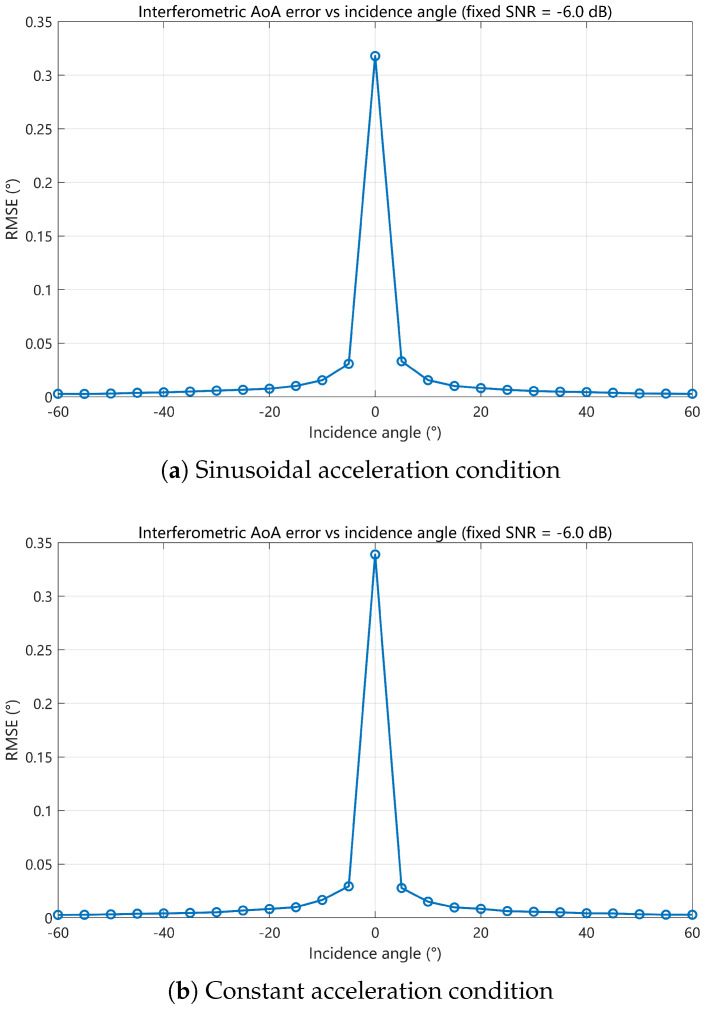
Comparison of interferometric angle estimation RMSE vs. incidence angle under a fixed SNR of −6 dB.

At a fixed SNR of −6 dB, Figure 12 shows the RMSE as a function of incident angle. Over most of the angular range, the estimation error remains below $0.05^{{}^{\circ}}$. A localized sensitive region appears near an incident angle of $0^{{}^{\circ}}$, where the phase differences approach ambiguity boundaries and noise perturbations may cause occasional ambiguity misclassification.

**Figure 13:**
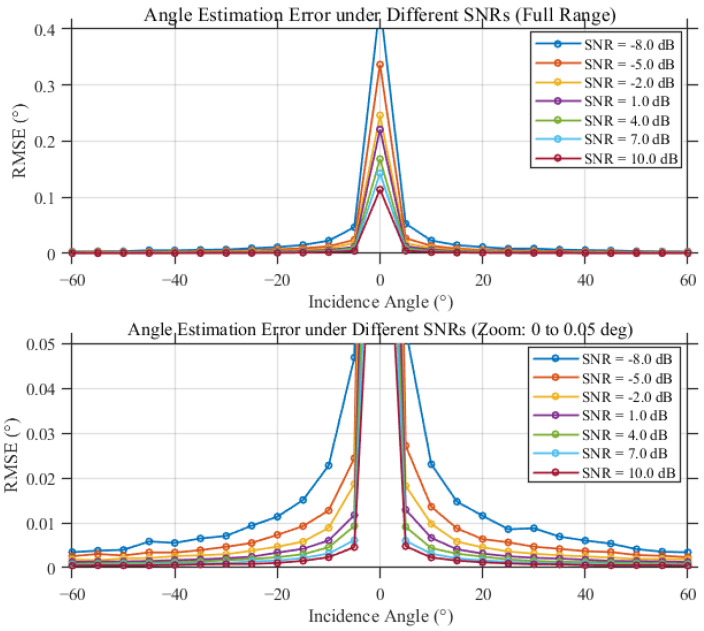
Time-modulated interferometric angle RMSE under different SNR levels.

**Figure 14:**
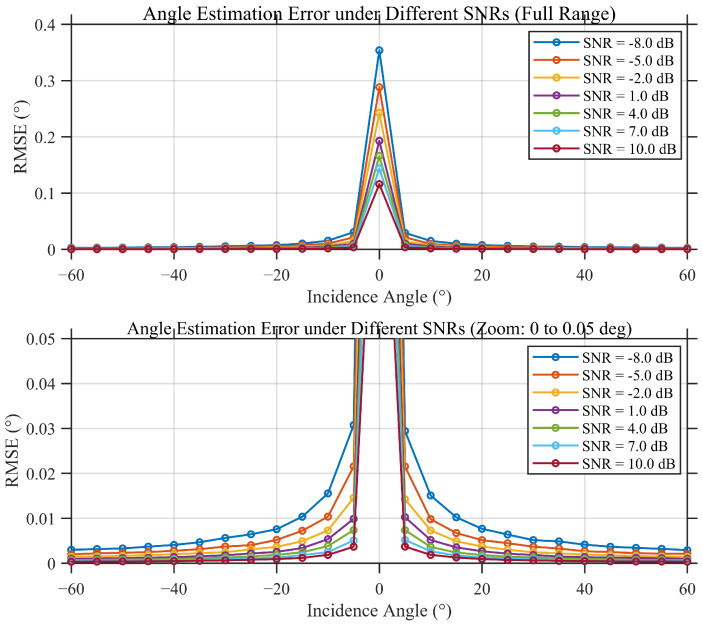
Continuous angle-estimation interferometric angle RMSE under different SNR levels.

Finally, Figure 13 and Figure 14 demonstrate that the proposed time-modulated scheme achieves performance comparable to the ideal continuous-reception baseline across both SNR and incident-angle domains. The primary discrepancy between the two schemes is confined to the near-zero-angle sensitive region, confirming the effectiveness and engineering feasibility of the proposed estimator under time-slotted sampling and single-channel reception constraints.

To further evaluate the applicability of the proposed angle estimation framework in more complex operational scenarios, a multi-target angle estimation experiment is conducted. In this simulation, multiple targets with distinct incident angles are simultaneously present within the field of view, and the long-baseline interferometric angle estimation is performed for each target.

**Figure 15:**
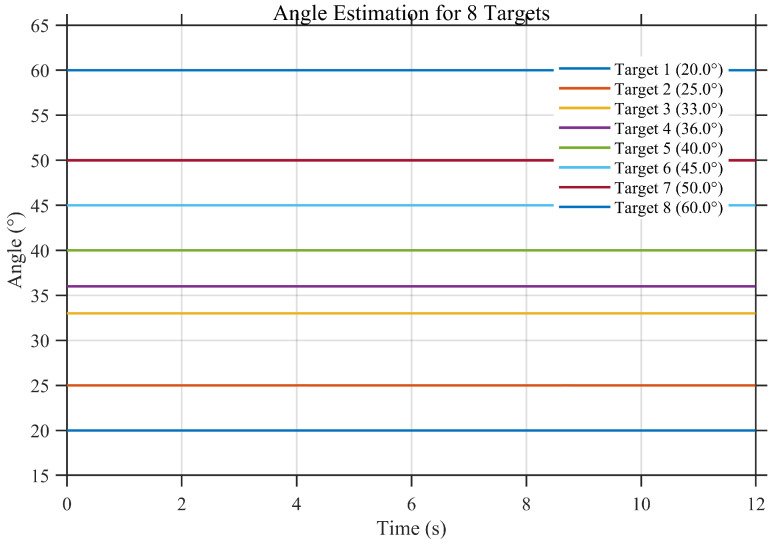
Interferometric angle estimation results under multi-target conditions.

Figure 15 presents the time histories of the estimated angles for multiple targets. It can be observed that the estimated angles closely follow the corresponding true angles throughout the observation interval, with no evident cross-target interference or tracking instability. This indicates that the proposed method is capable of resolving and tracking multiple targets simultaneously while maintaining estimation consistency.

**Figure 16:**
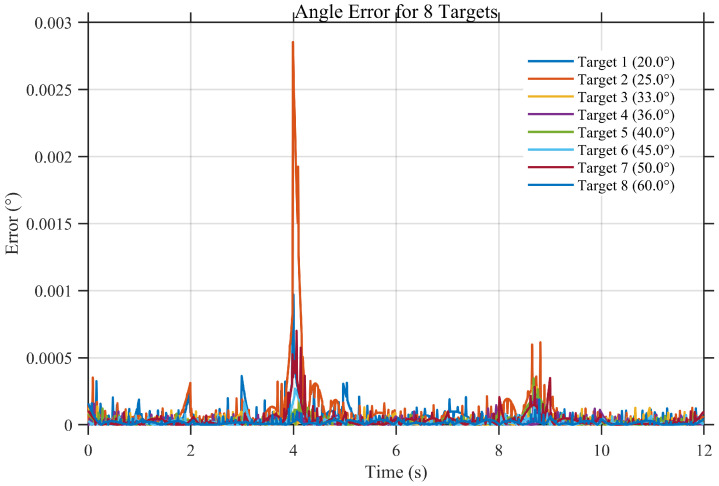
Time series of angle estimation errors under multi-target conditions.

Figure 16 further shows the corresponding angle estimation errors for all targets. The error magnitudes remain at low levels and fluctuate around zero without noticeable bias accumulation or long-term drift. Although occasional transient fluctuations are observed, the overall error behavior remains stable across different targets, demonstrating that the proposed method preserves high estimation accuracy under multi-target conditions.

These results confirm that the proposed interferometric angle estimation framework is not limited to single-target scenarios but can be effectively extended to multi-target applications. This capability is particularly important for practical SSPS and MWPT systems, where multiple receiving points or distributed targets may coexist within the coverage area.

### 5.4. Simulation Analysis of the Energy Centroid-Based Angle Estimation Method

**Figure 17:**
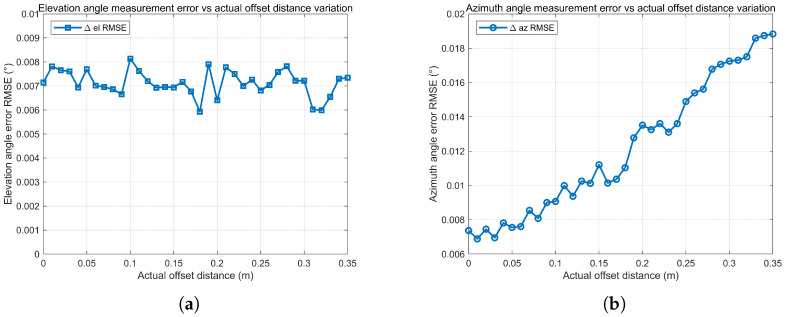
Angle estimation RMSE vs. actual energy centroid offset distance. (**a**) Azimuth angle RMSE vs. actual offset distance; (**b**) Elevation angle RMSE vs. actual offset distance.

**Figure 18:**
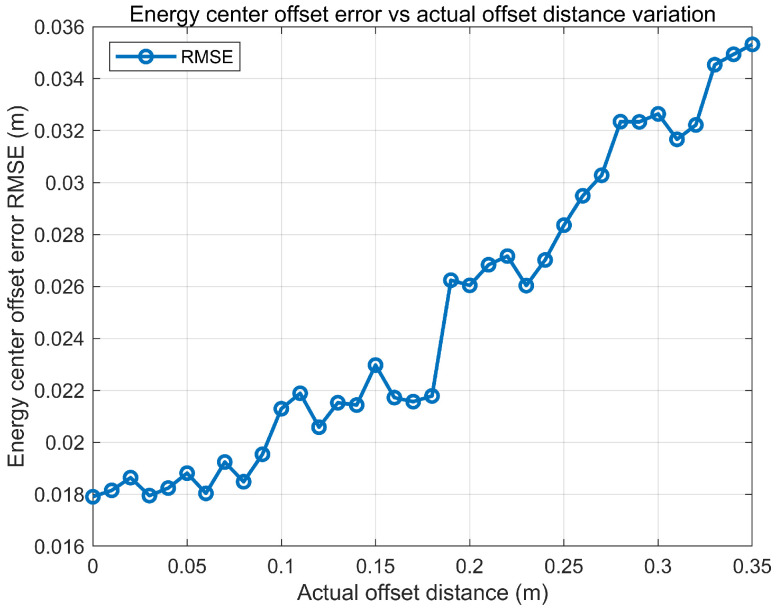
RMSE of estimated energy centroid offset distance vs. actual offset distance.

This section evaluates the energy centroid-based angle estimation method by scanning the true centroid offset on the receiving aperture and statistically analyzing the resulting angular errors. As shown in Figure 17, the elevation RMSE remains relatively stable over different offset conditions while the azimuth RMSE exhibits a mild increasing trend as the offset grows, indicating higher sensitivity to power distribution distortion in the azimuth direction. Figure 18 presents the RMSE of the estimated centroid offset distance, which increases moderately with the true offset but remains within a limited range.

Overall, under moderate-to-high SNR conditions, the energy centroid-based method provides stable low-rate angular observations, with typical accuracy of approximately $0.01^{{}^{\circ}}$ in elevation and $0.02^{{}^{\circ}}$ in azimuth. These characteristics make it well suited as a steady-state correction source within the proposed fusion framework.

### 5.5. Comparison of Interferometric Angle Estimation, Energy Centroid Measurement, and Multi-Source Fusion

**Figure 19:**
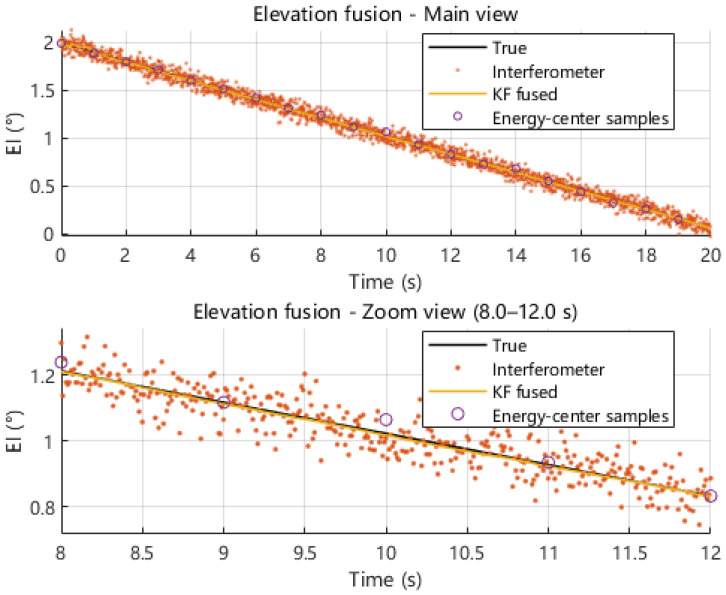
Comparison of azimuth angle estimates obtained from interferometric measurement, energy centroid measurement, and multi-source fusion.

**Figure 20:**
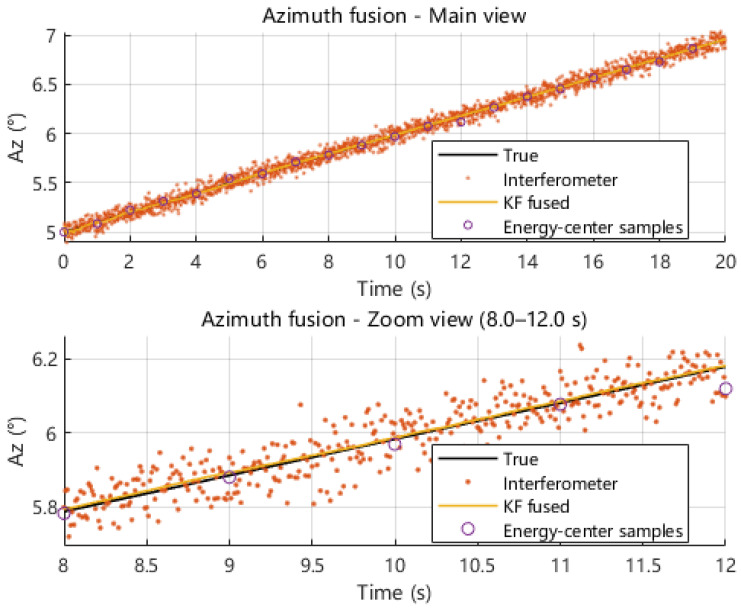
Comparison of elevation angle estimates obtained from interferometric measurement, energy centroid measurement, and multi-source fusion.

Under the same dynamic pointing scenario, we next compare the azimuth and elevation angle estimates obtained from interferometric measurement, energy centroid measurement, and Kalman-filter-based fusion. As shown in Figure 19 and Figure 20, the interferometric solution follows the true azimuth and elevation trajectories with a high update rate but exhibits noticeable high-frequency fluctuations, whereas the energy centroid-based solution provides sparse but smoother samples due to its 1 s update interval. By exploiting the complementary behaviors of these two modalities, the fused solution closely tracks the true trajectories while suppressing short-term jitter. The zoomed-in views further illustrate that fusion reduces local oscillations and maintains continuity without introducing obvious lag.

**Figure 21:**
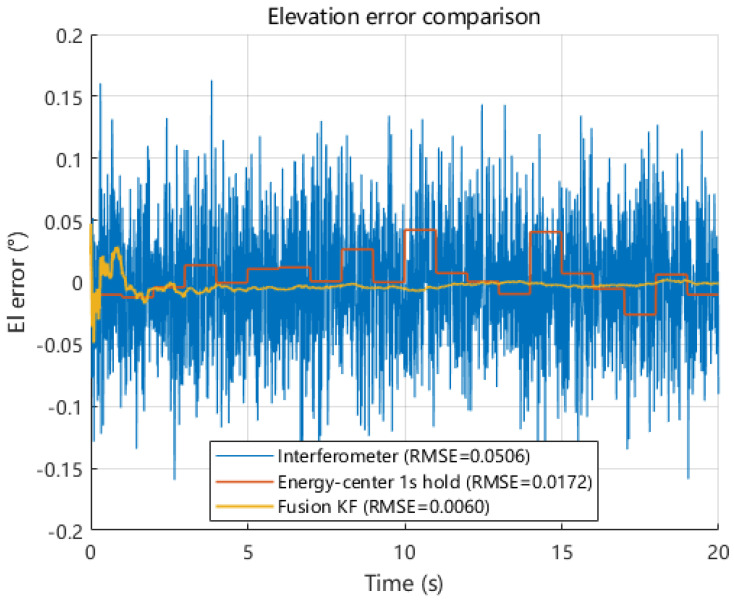
Time-domain error comparison of azimuth angle estimates under interferometric measurement, energy centroid measurement, and multi-source fusion.

**Figure 22:**
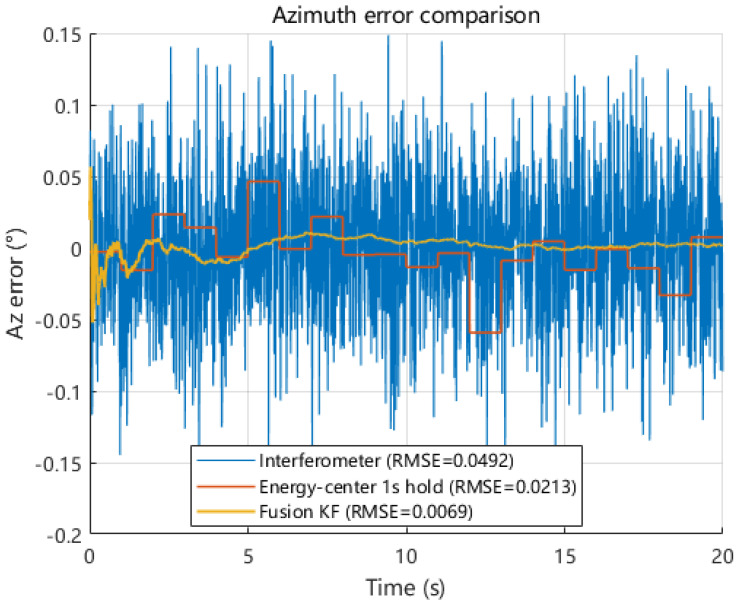
Time-domain error comparison of elevation angle estimates under interferometric measurement, energy centroid measurement, and multi-source fusion.

The corresponding time-domain error series are presented in Figure 21 and Figure 22. The interferometric errors show dense high-frequency variations, while the energy centroid errors remain relatively small but appear as step-like segments between updates. In contrast, the fusion output exhibits the smallest fluctuation range in both azimuth and elevation. Quantitatively, the fusion RMSE reaches $0.0069^{{}^{\circ}}$ in azimuth and $0.0060^{{}^{\circ}}$ in elevation, compared with $0.0492^{{}^{\circ}}$ and $0.0506^{{}^{\circ}}$ for interferometric estimation and $0.0213^{{}^{\circ}}$ and $0.0172^{{}^{\circ}}$ for energy centroid measurement. These results demonstrate that heterogeneous measurement fusion can simultaneously improve accuracy and temporal smoothness for both azimuth and elevation under dynamic conditions.

Quantitative results are summarized in Table 3. Multi-source fusion reduces the azimuthal RMSE from $0.0492^{{}^{\circ}}$ to $0.0069^{{}^{\circ}}$ and the elevation RMSE from $0.0506^{{}^{\circ}}$ to $0.0060^{{}^{\circ}}$ compared with interferometric estimation alone. It also achieves lower RMSE than energy centroid measurement while preserving continuous high-rate outputs. These results confirm that heterogeneous measurement fusion improves angle estimation accuracy in both azimuth and elevation under dynamic conditions. The resulting RMSE levels are used in the following section to quantify the system-level impact of pointing errors on microwave power transmission efficiency.

### 5.6. Simulation Analysis of the Impact of Angle Estimation Errors on Power Transmission Efficiency

**Figure 23:**
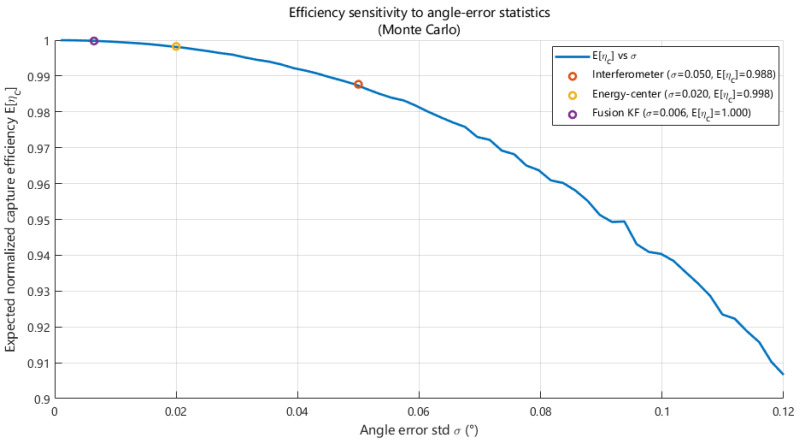
Statistical sensitivity curve of expected normalized capture efficiency vs. angle error standard deviation.

In the efficiency analysis, the angular error is modeled as a zero-mean Gaussian random variable, which is a common assumption in beam-pointing error analysis and tracking performance evaluation. This assumption provides a statistically representative model for small-angle pointing errors and enables tractable Monte Carlo performance analysis. To establish a quantitative link between angle estimation accuracy and system-level power transmission performance, this study analyzes the impact of beam pointing errors on capture efficiency based on the aperture coupling model and the normalized capture efficiency definition in [33]. Under the small-angle assumption, pointing errors can be equivalently interpreted as lateral displacement of the energy beam footprint on the receiving aperture, leading to power truncation and efficiency degradation. Figure 23 illustrates the statistical sensitivity of the expected capture efficiency to the angle error standard deviation under a Gaussian error assumption.

By substituting the RMSE levels listed in Table 3 into the sensitivity curve in Figure 23, the expected capture efficiencies corresponding to the three angle estimation schemes are obtained. Specifically, interferometric angle estimation with an error level of approximately $0.05^{{}^{\circ}}$ yields an expected capture efficiency of about 0.988. The energy centroid-based method, with an error level of approximately $0.02^{{}^{\circ}}$, improves the expected efficiency to about 0.998. The multi-source fusion output, achieving an angle error level of approximately $0.006^{{}^{\circ}}$, results in a capture efficiency close to unity. These results indicate that improving angular accuracy from approximately $0.05^{{}^{\circ}}$ to about $0.006^{{}^{\circ}}$ can significantly reduce efficiency loss caused by beam pointing errors.

**Figure 24:**
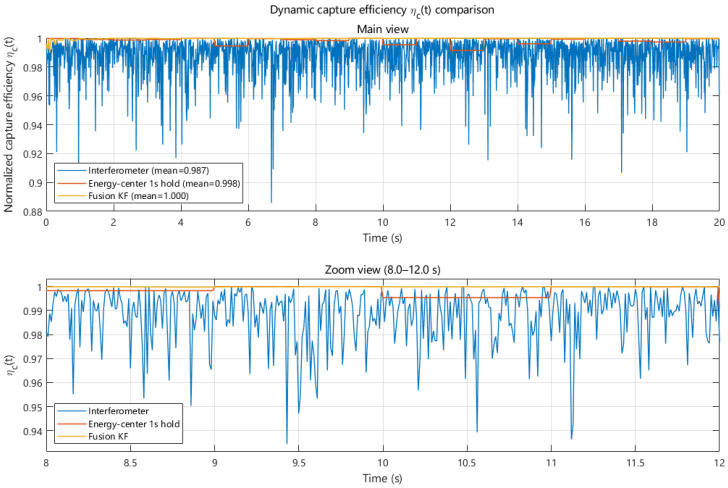
Dynamic capture efficiency comparison under different angle estimation methods.

For engineering-oriented accuracy allocation, Table 4 summarizes the maximum allowable angle error standard deviation and the corresponding equivalent lateral displacement under different capture efficiency constraints, providing a quantitative basis for translating efficiency requirements into measurement performance specifications. Furthermore, Figure 24 compares the capture efficiency time series driven by the three angle estimation outputs. The interferometric output exhibits frequent instantaneous efficiency drops due to high-frequency noise. The energy centroid-based output is smoother overall, but shows holding characteristics limited by its update interval. In contrast, the fusion-based approach suppresses high-frequency fluctuations while achieving the highest average efficiency and the smallest variation, thereby improving both power transmission efficiency and stability under dynamic conditions.

## 6. Conclusions

To address the stringent high-precision pointing requirements in space solar power-based microwave wireless power transmission scenarios, this paper establishes a quantitative model that characterizes the impact of beam pointing errors on received power and capture efficiency. A dual-chain angle measurement framework is proposed by combining interferometric angle estimation with energy field reconstruction. In the proposed framework, the guide-link interferometric chain enables high-rate angle updates to support dynamic tracking while the energy-link chain reconstructs the received power distribution to extract the energy centroid offset, providing high-accuracy and stable angle corrections.

By jointly exploiting these two complementary measurement mechanisms, a multi-source fusion scheme based on Kalman filtering is developed to achieve continuous, smooth, and robust angle estimation under dynamic conditions. With an interferometric angle error standard deviation of approximately $0.05^{{}^{\circ}}$, an energy centroid-based measurement accuracy of approximately $0.02^{{}^{\circ}}$, and an energy centroid update interval of 1s, the simulation results demonstrate that our fusion approach achieves RMSEs of approximately $0.0069^{{}^{\circ}}$ and $0.0060^{{}^{\circ}}$ in azimuth and elevation, respectively. Compared with interferometric estimation alone, the proposed fusion method significantly suppresses high-frequency angle fluctuations while maintaining continuous output, thereby improving both dynamic tracking performance and steady-state accuracy.

Furthermore, a capture-efficiency sensitivity model is established on the basis of the energy distribution on the receiving aperture in order to quantify the relationship between angle estimation errors and power transmission efficiency. Sensitivity analysis reveals a monotonic decrease in the expected capture efficiency $E[\eta_{c}]$ as the angle error standard deviation increases. The corresponding expected capture efficiencies for the angle estimation outputs based on the interferometric, energy centroid, and fusion approaches are approximately 0.988, 0.998, and close to unity, respectively. Dynamic efficiency simulations further show that the fusion-based approach effectively reduces efficiency fluctuations and maintains higher average capture efficiency under time-varying conditions.

## Figures and Tables

**Table 1:** System and signal processing parameters.

Parameter	Value
RF carrier frequency	32 GHz
IF frequency	70 MHz
BOC subcarrier frequency	30.69 MHz
Spreading code rate	1.023 MHz
Sampling frequency	200 MHz
Initial Doppler frequency	100 kHz
FLL loop bandwidth	20 Hz
PLL loop bandwidth	10 Hz
DLL bandwidth	2 Hz
DLL early–late correlator spacing	0.5 chips
Loop update interval	0.4 ms
Long baseline length	0.317 m
Short baseline length	0.139 m
Transmission distance	500 m
Angle measurement range	$-60^{{}^{\circ}}$ to $60^{{}^{\circ}}$
Receiving aperture size	6m×6m
Energy-centroid update interval	1 s

**Table 2:** Modeling parameters for Kalman fusion simulations.

Parameter	Value
State vector	${\left[\alpha ,\;\beta ,\;\dot{\alpha },\;\dot{\beta }\right]}^{T}$
State model	Constant-velocity model
Filter update interval	0.01s
Noise model	Zero-mean Gaussian noise
Interferometric update rate	100Hz
Energy-centroid update rate	1Hz
Interferometric measurement std	$\sigma_{\mathrm{int}}=\left[0.05^{{}^{\circ}},\;0.05^{{}^{\circ}}\right]$
Energy-centroid measurement std	$\sigma_{\mathrm{ec}}=\left[0.02^{{}^{\circ}},\;0.02^{{}^{\circ}}\right]$
Interferometric covariance	$R_{I}=\mathrm{diag}\left(0.05^{2},\;0.05^{2}\right)$
Energy-centroid covariance	$R_{E}=\mathrm{diag}\left(0.02^{2},\;0.02^{2}\right)$
Angular acceleration std	$0.005^{{}^{\circ}}$/$s^{2}$
Process covariance	$Q=G\;\mathrm{diag}\left(\sigma_{a,\alpha }^{2},\sigma_{a,\beta }^{2}\right)\;G^{T}$
Monte Carlo runs (RMSE curves)	100
Monte Carlo runs (efficiency sensitivity)	3000

**Table 3:** RMSE comparison of different angle estimation methods.

Method	Azimuth RMSE (°)	Elevation RMSE (°)
Interferometric estimation	0.0492	0.0506
Energy-centroid measurement (1 s update)	0.0213	0.0172
Multi-source fusion (KF)	0.0069	0.0060

**Table 4:** Maximum allowable angle error standard deviation derived from capture efficiency constraints.

Capture Efficiency Constraint $\eta_{\mathrm{th}}$	Maximum Allowable $\sigma_{\max}$ (°)	Equivalent Lateral Displacement $d_{\mathrm{rms}}$ (m)
0.999	0.0148	0.1291
0.998	0.0207	0.1804
0.995	0.0319	0.2781
0.990	0.0446	0.3894
0.980	0.0615	0.5370
0.950	0.0910	0.7943
0.900	0.1200	1.0472

## Data Availability

Due to confidentiality restrictions, the datasets used in this study are not publicly available. Partial datasets may be provided by the corresponding author upon reasonable request.

## Abbreviations

The following abbreviations are used in this manuscript:

AoA	Angle of Arrival
BOC	Binary Offset Carrier
DLL	Delay-Locked Loop
DoA	Direction of Arrival
FLL	Frequency-Locked Loop
IF	Intermediate Frequency
KF	Kalman Filter
MWPT	Microwave Wireless Power Transfer
NCO	Numerically Controlled Oscillator
PLL	Phase-Locked Loop
RF	Radio Frequency
RMSE	Root Mean Square Error
SNR	Signal-to-Noise Ratio
SSPS	Space Solar Power Station/System
